# Profiles of depression in a treatment‐seeking Hispanic population: Psychometric properties of the Patient Health Questionnaire‐9

**DOI:** 10.1002/mpr.1851

**Published:** 2020-08-30

**Authors:** Michael O. Killian, Katherine Sanchez, Brittany H. Eghaneyan, Leopoldo J. Cabassa, Madhukar H. Trivedi

**Affiliations:** ^1^ College of Social Work Florida State University Tallahassee Florida USA; ^2^ School of Social Work University of Texas at Arlington Arlington Texas USA; ^3^ Department of Psychiatry UT Southwestern Medical Center Dallas Texas USA; ^4^ Department of Social Work California State University, Fullerton Fullerton California USA; ^5^ George Warren Brown School of Social Work Washington University in St. Louis St. Louis Missouri USA

**Keywords:** depression, Hispanics, latent profile analysis, primary care

## Abstract

**Objectives:**

Screening instruments can be powerful tools in assisting primary care providers with detecting depression in their patients and monitoring treatment response. Health disparities among racial and ethnic minorities result from inaccurate assessment in primary care.

**Methods:**

The current study used baseline data from two federally funded research studies of treatment for depression among Hispanics in primary care. The Patient Health Questionnaire‐9 (PHQ‐9) was administered at baseline prior to the study interventions, and 499 participants provided responses.

**Results:**

Confirmatory factor analyses found excellent factor validity for the PHQ‐9, yet reliability remained poor. Possible heterogeneity in depressive item scores was examined, and latent profile analysis identified four distinct profiles of PHQ‐9 responses. Profiles included a lower depression, moderate/somatization, moderate/negative self‐view, and severe depression profiles. Results indicate modest support for the PHQ‐9 and its use among Hispanics for the purpose of depression screening.

**Conclusion:**

Capturing four profiles of depression in a large primary care sample helps characterize the manifestation of depression in a Hispanic population. The single item related to fatigue had the greatest variation across groups indicating it might be useful as a screening item. Inadequate evaluation of symptoms could lead to significant under identification of the disorder among Hispanics.

**Abbreviations**
AIC: Akaike Information Criteria
BIC: Bayesian Information Criteria
BLRT: Bootstrap Likelihood Ratio Test
CFA: confirmatory factor analysis
CFI: Comparative Fit Index
DCM: Depression Care Manager
DEI: Depression Education Intervention
DESEO: Depression Screening and Education: Options to Reduce Barriers to Treatment
DSM: Diagnostic and Statistical Manual of Disorders
FQHC: Federally Qualified Health Center
GAD‐7: Generalized Anxiety Disorder anxiety measure
IC: information criteria
LCSW: Licensed Clinical Social Worker
LMR: Lo–Mendell–Rubin test
LPA: latent profile analysis
LVMM: latent variable mixture modeling
MDD: major depressive disorder
METRIC: Measurement, Education and Tracking in Integrated Care
PHQ‐9: Patient Health Questionnaire‐9
PTSD: post‐traumatic stress disorder
RMSEA: root‐mean‐square error of approximation
SRMR: weighted root‐mean‐square residual
SSA‐BIC: sample‐size adjusted BIC
TLI: Tucker–Lewis index
U.S.: United States
WLSMV: weighted least square mean and variance adjusted

## 1 INTRODUCTION

Hispanic patients with depression and dysthymia have disproportionately high numbers of somatic symptoms, particularly among women less than 40 years of age (Chong, Reinschmidt, & Moreno, 2010). Within primary care, vague or unexplained symptoms such as aches, pain and fatigue are often presenting symptoms of depression (Trivedi, 2004). The lifetime prevalence of psychiatric disorders among Hispanics has been estimated to be 28.1% for men and 30.2% for women (Alegria et al., 2007), while overall prevalence of depression is estimated to be 27.0% (Wassertheil‐Smoller et al., 2014). Hispanic patients with comorbid depression and chronic disease inadvertently delay intervention because of a somatic presentation of symptoms, which impedes accurate and timely detection of depression (Eghaneyan, Sanchez, & Mitschke, 2014; Interian et al., 2010). Such delays in addressing the underlying depression can not only make remission difficult but can make treatment of the physical condition challenging (Kravitz & Ford, 2008). Major depression increases the burden of chronic illness by increasing perception of physical symptoms, causing additional impairment in functioning (Katon, 2011; Unutzer, Schoenbaum, Druss, & Katon, 2006).

Self‐report depression screening and measurement instruments can be powerful tools in assisting primary care providers with detecting depression in their patient population, diagnosing depression and monitoring treatment response. These instruments can also help measure a patient's overall depression severity over time, as well as the specific symptoms that are improving or not with treatment (Trivedi, 2009). Accurate screening, diagnosis and treatment of depression are entirely dependent on accurate measurement of symptoms (Thielke, Vannoy, & Unutzer, 2007). Inaccurate assessment in primary care is an independent predictor of poor control of chronic disease and is a significant contributor to health disparities, lack of patient satisfaction and poor‐quality patient education and understanding of their disorder (Sanchez, Ybarra, Chapa, & Martinez, 2016).

A symptom‐level approach may provide important insight into how various individual factors relate to the broader disorder (Djelantik, Robinaugh, Kleber, Smid, & Boelen, 2019; Fried & Nesse, 2015). Researchers have increasingly used latent variable mixture modeling (LVMM; McCutcheon, 1987) techniques, person‐centered analytic approaches, to identify groups of patients with similar profiles of symptoms. Within LVMM, latent profile analysis (LPA) is able to use symptoms to estimate an individual's likely membership in differing profiles of symptoms and then test for differences in characteristics among the resulting profiles (e.g., demographics and differences in self‐report measure scores; Muthén & Muthén, 2000). Estimating possible heterogeneity in depressive symptoms and then grouping individuals through common profiles of responses allows for researchers and clinicians to understand differing clinical presentations of those seeking treatment. Analysis of symptom level associations between depression and other factors have previously suggested paths by which these factors affect or are affected by the broader syndrome and their relationship to other disorders such as grief and PTSD (Djelantik et al., 2019).

The central aims of the current study were to (1) evaluate the psychometric quality of the Patient Health Questionnaire‐9 (PHQ‐9) measure (Kroenke & Spitzer, 2002) through the use of confirmatory factor analysis (CFA) and (2) explore possible heterogeneity in the symptom‐level presentation of depression in a large sample of Hispanic primary care patients. The PHQ‐9 is the most commonly used depression screening and measurement tool in medical settings (Savoy & O'Gurek, 2016). We expect the unidimensional model of the PHQ‐9 to be supported in the current sample. A previous study of the PHQ‐9 in a large sample of educated Mexican women (*n* = 55,555; 89.4% with a university degree or more) found support for the PHQ‐9 as a unidimensional model (Familiar et al., 2015). In the current analyses, we used CFA to evaluate the factor validity of the PHQ‐9 followed by a three‐step LPA approach which included the estimated profiles of depressive symptoms, posterior probabilities of membership, and classification uncertainty rates. The specific three‐step LPA approach used predictors of latent profile membership yet included an estimation posterior probability and classification uncertainty rates into the full model with predictors of profile membership (Asparouhov & Muthén, 2013; Vermunt, 2010).

## 2 METHODS

### 2.1 Study design and setting

Data for the current study was baseline data collected as part of two federally funded intervention studies of Hispanic patients who screened positive for depression in primary care. The first study, *Depression Screening and Education: Options to Reduce Barriers to Treatment* (DESEO), was a one‐group pretest–posttest design assessing a culturally‐adapted Depression Education Intervention's (DEI) effects on depression knowledge, stigma, and engagement in treatment (Sanchez, Eghaneyan, & Trivedi, 2016). The second study, *Measurement, Education and Tracking in Integrated Care* (METRIC), was a randomized controlled trial testing the effectiveness of a culturally appropriate DEI, and included a collaborative model and culturally tailored care management strategies, including a bilingual, Licensed Clinical Social Worker (LCSW) serving as a Depression Care Manager (Sanchez, Eghaneyan, Killian, Cabassa, & Trivedi, 2017). Informed by DESEO, the aims of METRIC were to increase knowledge about depression and reduce stigma about treatment while increasing engagement in care among Hispanics by testing a culturally appropriate depression education *fotonovela* developed by Cabassa, Molina, and Baron (2012).

Each study took place in a Federally Qualified Health Center (FQHC) in north Texas between February 2015 and February 2018. FQHCs are community‐based health care providers that provide primary care services in underserved areas and meet stringent set of requirements including offering sliding scale fees and operating under a board that includes patients (Health Resources & Services Administration, 2018). The community clinics where DESEO and METRIC took place were a part of the same non‐profit health care organization that provides comprehensive primary care and preventive services including family medicine, pediatrics, and obstetrics/gynecology to a primarily low‐income, Hispanic population. The studies were reviewed and approved by the Institutional Review Board of the University of Texas at Arlington.

### 2.2 Participants

All adult primary care patients were universally screened for depression using the PHQ‐9 measure (Kroenke & Spitzer, 2002) delivered in English or Spanish, depending on the patient's preference, during annual or new/non‐acute visits as part of normal clinical practice. When a patient scored a five or more on the PHQ‐9, the primary care provider would initiate a “warm hand off”, wherein the patient was referred and introduced to the clinic's bilingual LCSW. Once referred, the LCSW assessed patients for the presence of the nine diagnostic criteria for major depression from the Diagnostic and Statistical Manual of Disorders (DSM‐IV; American Psychiatric Association, 1994) through a clinical interview.

Patients who met the following inclusion criteria were invited to participate in the studies: 18 years or older, self‐identified as Hispanic, met diagnostic criteria for major depressive disorder, and were not already receiving treatment for depression (medication and/or psychotherapy). During the screening process, all patients were also given the Generalized Anxiety Disorder‐7 (GAD‐7) anxiety measure (Spitzer, Kroenke, Williams, & Lowe, 2006). All participants signed informed consent prior to participation. The total sample for the current analysis included baseline data from the DESEO (*N* = 349) and METRIC (*N* = 150) samples for a combined total of 499 participants. In both studies, all measures were completed prior to receipt of the educational intervention.

### 2.3 Measures

#### 2.3.1 Depression

Depression severity was measured using the PHQ‐9, a self‐report measure that assesses the frequency of depression symptoms within the last 2 weeks using each of the of the nine DSM‐IV criteria for depression. Total scores range from 0 to 27 with scores of 5–9 representing mild depression, 10–14 representing moderate depression, 15–19 representing moderately severe depression, and greater than or equal to 20 representing severe depression. The PHQ‐9 has demonstrated to be a reliable and valid measure of depression severity in racially and ethnically diverse primary care samples and is available in both English and Spanish (Huang, Chung, Kroenke, Delucchi, & Spitzer, 2006). Studies examining the factor structure of the PHQ‐9 have demonstrated support for the measure as a single‐factor structure among both English and Spanish speaking Hispanics with strong internal consistency (Fried & Nesse, 2015; Huang et al., 2006).

#### 2.3.2 Anxiety

Anxiety symptom severity was measured using the GAD‐7, a 7‐item self‐report scale for identifying the presence of generalized anxiety disorder (Spitzer et al., 2006). The items of the GAD‐7 assess frequency of symptoms over the last 2 weeks based on the diagnostic criteria for generalized anxiety disorder in the DSM‐IV, with responses ranging from 0 for “not at all” to 3 for “nearly every day.” The GAD‐7 has been found to be a reliable and valid measure for use with Hispanic Americans and has demonstrated strong internal consistency reliability for both the English and Spanish versions (Mills et al., 2014).

#### 2.3.3 Demographics

Demographic measures were collected via self‐report and medical record extraction and included age at enrollment, gender, primary language, marital status, and education level.

### 2.4 Statistical analyses

#### 2.4.1 CFA of PHQ‐9

CFA was used to assess the fit of the PHQ‐9 to the baseline DESEO and METRIC data (*N* = 499). CFA modeling used mean‐ and variance‐adjusted weighted least squares estimator in Mplus 8.1 (Muthén & Muthén, 2018). Due to the clinical setting and nature of data collection supported by clinical staff in DESEO and METRIC, the PHQ‐9 scores had no missing data. The model chi‐square (*χ* ^2^) value (Kline, 2011), model chi‐square value per degrees of freedom (*χ* ^2^/df; Bollen, 1989), and root‐mean‐square error of approximation (RMSEA; Hu & Bentler, 1999) with a 90% confidence interval (90% CI) were used to assess model fit. Good model fit was indicated by a lower and non‐significant *χ* ^2^ value, *χ* ^2^/df value less than 3.0, and RMSEA scores less than 0.08 or 0.10. Additional fit indices included the Bentler Comparative Fit Index (CFI), Tucker–Lewis index (TLI), and weighted root‐mean‐square residual (SRMR; Brown, 2006). CFI and TLI scores less than 0.95 and a SRMR less than 0.10 indicate model fit with the data. Modifications to the model were included where correlation of error terms would generate a model *χ* ^2^ difference greater than 9.0. Additional analyses included tests of reliability and psychometric validity and were performed in SPSS 25.0.

#### 2.4.2 Latent profile analysis

Self‐reported items from the PHQ‐9 were used as indicators in a LPA model using Mplus 8.2. The fit of the LPA model to the data was assessed through several fit indices and based on LVMM reporting recommendations of Nylund, Asparouhov, and Muthén (2007). Log likelihood (Lanza, Flaherty, & Collins, 2003) and information criteria based fit statistics were used to assess model fit. Lower values of the Bayesian Information Criteria (BIC; Schwartz, 1978), Akaike Information Criteria (Akaike, 1987), and Sample‐Size Adjusted BIC (Sclove, 1987) each indicate better fit and model parsimony (Muthén & Muthén, 2003). The Lo–Mendell–Rubin test (LMR; Lo, Mendell, & Rubin, 2001) and the parametric Bootstrap Likelihood Ratio Test (BLRT; McLachlan & Peel, 2000) are both log likelihood ratio tests which compare these values between the model with *k* profiles and a model with one fewer profiles, or *k*−1 (Bollen, 1989).

In the case of LPA, profile membership has traditionally been imputed from the maximum‐probability assignment rule (Nagin, 2005) in which individuals are categorized into a profile based on their greatest posterior probability, the likelihood of membership in a particular profile for all within a sample (Muthén, 2001). Traditionally in subsequent analyses, profile membership is then treated as a categorical, observed variable. However, problems may arise from this type of discrete categorization (Asparouhov & Muthén, 2013; Lanza, Tan, & PBray, 2013). Profile membership determined by greatest individual posterior probability ignores the uncertainty of membership for individuals in the final LPA model. Treating probability scores as simple categories introduces measurement error within future analysis. Collapsing probability scores into a categorical variable may attenuate the relationship between profiles and other explanatory or outcome variables, especially when other variables are expected to have significant associations with the indicators of profiles used in the initial LPA modeling.

The LMR and BLRT tests produce a *p*‐value testing the value of the *k* model against a model with one fewer profile. A significant *p*‐value would indicate the value of an additional profile in the *k* model over that of a more parsimonious model. Finally, entropy scores are the average classification probabilities for each individual's most probable profile in the model (Celeux & Soromenho, 1996), which indicate the accuracy of profile classification. Entropy scores range from 0.00 to 1.00 with scores greater than 0.80 or 0.90 indicating more accurate classification (Weden & Zabin, 2005). A model entropy score of 1.00 would indicate perfect probability scores for profile membership. Furthermore, the sample size of 499 meets current suggestions for LPA modeling. Though no clear requirements for sample size exist in LVMM (Muthén & Muthén, 2002), Berlin, Williams, and Parra (2014) suggest sufficient sample size to avoid convergence problems during modeling and difficulty identifying smaller, yet meaningful, classes or profiles in the sample. More recently, sample sizes of at least 300 have been recommended (Methodology Center at Pennsylvania State University, 2018).

## 3 RESULTS

### 3.1 Demographic characteristics

The sample of 499 Hispanics screening positive for depression across two samples (Table 1) included 459 women (92.0%). The mean age of the sample was 39.02 ± 10.13 years. Nearly all were Spanish‐speaking (*n* = 467, 93.8%). Most were either married or cohabiting with a partner (*n* = 352, 71.0%). Educational attainment among the sample was low with a majority not completing a high school education (*n* = 297, 59.4%). The sample had 354 participants (70.8%) reporting either moderately severe (PHQ‐9 scores of 15–19) or severe (PHQ‐9 scores of 20–27) depression. Overall mean PHQ‐9 scores were 16.88 ± 4.09, and mean GAD‐7 anxiety scores were 12.85 ± 4.63.

**TABLE 1:** Descriptive statistics of sample

Demographic and patient characteristic	Total sample (*N* = 499)
Age, *M* ± *SD*	39.02 ± 10.13
Gender, female, *n* (%)	459 (92.0%)
Spanish speaking, yes, *n* (%)	467 (93.8%)
Marital status, *n* (%)	
Married/cohabitating	352 (71.0%)
Never married	51 (10.3%)
Widowed	10 (2.0%)
Divorced	50 (10.1%)
Other	33 (6.6%)
Education level, *n* (%)	
8th grade or less	182 (37.2%)
Some high school	115 (23.5%)
High school or General Educational Development Test (GED)	126 (25.8%)
Vocational or trade school	10 (2.0%)
Some college	40 (8.2%)
College degree	15 (3.1%)
Patient Health Questionnaire‐9, *M* ± *SD*	16.88 ± 4.09
Minimal depression (score 0–4), *n* (%)	0 (0%)
Mild depression (score 5–9), *n* (%)	10 (2.0%)
Moderate depression (score 10–14), *n* (%)	135 (27.1%)
Moderately severe depression (score 15–19), *n* (%)	215 (43.1%)
Severe depression (score 20+), *n* (%)	139 (27.9%)
Generalized Anxiety Disorder‐7 anxiety scores, *M* ± *SD*	12.85 ± 4.63

### 3.2 CFA of PHQ‐9

PHQ‐9 scores were assessed through item‐total correlations followed by CFA modeling. PHQ‐9 item scores correlated significantly with scale total scores, ranging from 0.422 to 0.596, all *p* < 0.001. Initial CFA analyses indicated moderate fit of the model (*χ* ^2^ [27, *N* = 499] = 99.685, *p* < 0.001; *χ* ^2^/df = 3.69; CFI = 0.925; TLI = 0.900; RMSEA = 0.073 [90% CI: 0.058, 0.089]; SRMR = 0.046).

**FIGURE 1:**
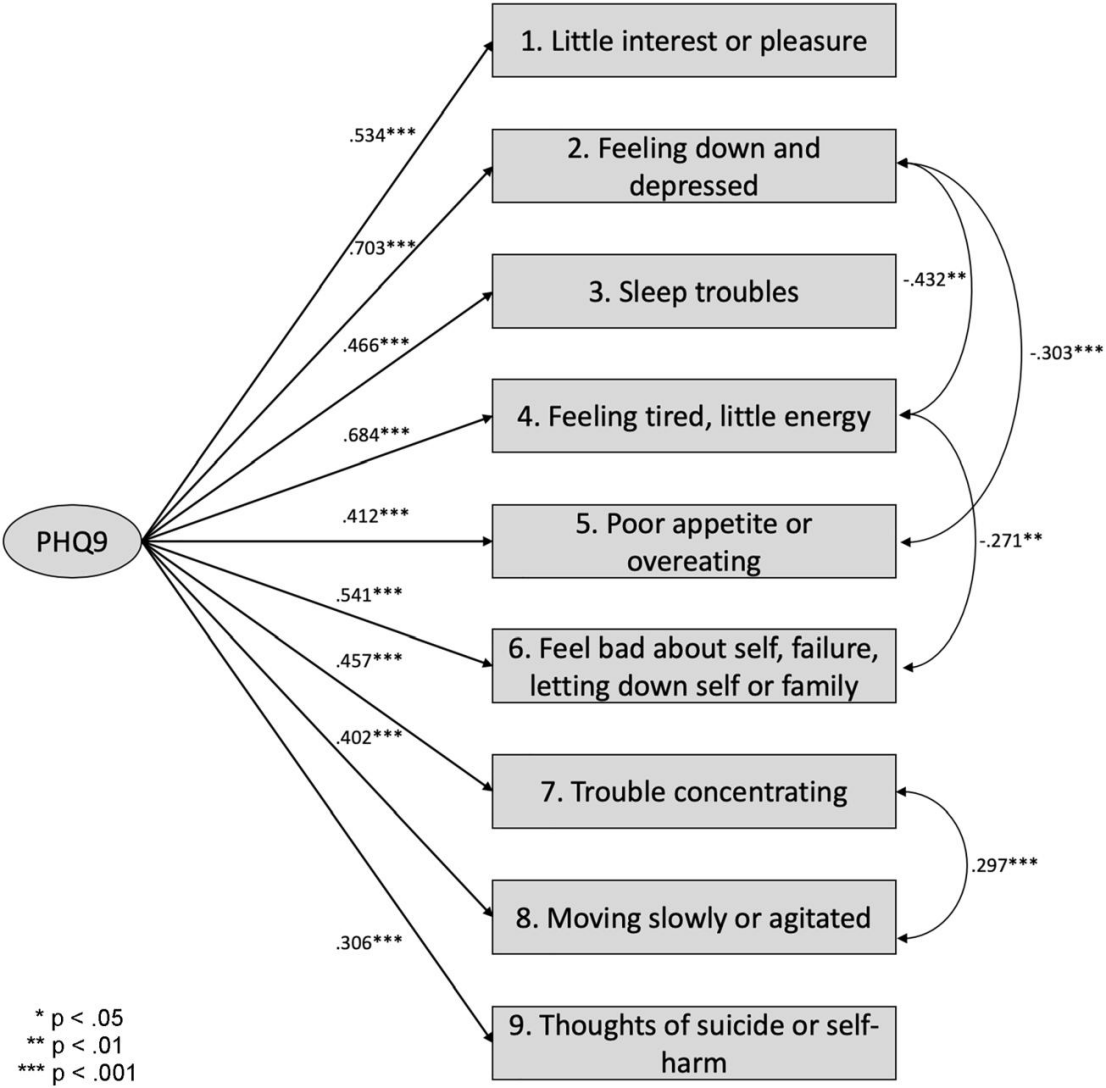
Patient Health Questionnaire‐9 (PHQ‐9) confirmatory factor analysis (CFA) diagram (*N* = 499). This figure presents the CFA final model for the PHQ‐9 with the sample (*N* = 499). Values along the straight lines represent standardized factor loadings flagged for levels of significance. Values along curved lines represent correlated error terms included in the model following modifications. *p < 0.05; **p < 0.01; ***p < 0.001

Four correlated error terms were added to the model resulting in excellent model fit for the PHQ‐9 (χ^2^ [23, *N* = 499] = 33.313, *p* = 0.0758; χ^2^/df = 1.45; CFI = 0.989; TLI = 0.983; RMSEA = 0.030 [90% CI: 0.000, 0.051]; SRMR = 0.030). Standardized factor loadings ranged from 0.306 to 0.703, all *p* < 0.001 (Figure 1). Discriminant item functioning testing through corrected item‐total correlations indicated good to excellent discrimination among all items (*r* ranged from 0.232 to 0.425). Despite the support for factor validity for the measure in the current sample, internal consistency reliability remained poor (*α* = 0.679). Negative correlations between error variances of items were observed in the model and may have resulted in a lower internal consistency reliability coefficient.

### 3.3 Measurement validity of PHQ‐9

Construct and criterion‐related validity was assessed through testing the association between PHQ‐9 scores and other reported patient demographics and self‐report measure scores. PHQ‐9 scores were strongly and significantly correlated with GAD‐7 anxiety scores, *r* = 0.558, *p* < 0.001. Women in the sample reported significantly greater PHQ‐9 scores (*M* = 17.03, *SD* = 4.12) than men (*M* = 15.10, *SD* = 3.39, *t* = 2.89, df = 497, *p* = 0.004, Cohen *d* = 0.48). PHQ‐9 scores did not significantly differ by marital status (*F* = 0.640, *p* = 0.634) or educational level (*F* = 1.041, *p* = 0.393).

### 3.4 Latent depression profiles

#### 3.4.1 Initial LPA modeling

LPA modeling indicated a four‐profile solution best fit the data (Table 2) and was a significant improvement on a model with three profiles (LMR *p* = 0.0146; BLRT *p* < 0.001). Estimation of a model with five profiles resulted in a decrease in model quality and fit. Entropy scores (0.894) further supported the latent categorical variable and fit with the data with four profiles. Furthermore, profiles had varying patterns of responses across PHQ‐9 items satisfying a LVMM modeling concern reported by Morin and Marsh (2015).

**TABLE 2:** LPA model results using the PHQ‐9 item scores, continuous

Profiles	Log likelihood	AIC	BIC	Sample‐size adjusted BIC	Entropy	BLRT *p*‐value	LMR *p*‐value
1	−5643.245	11,322.489	11,398.316	11,341.183	‐	‐	‐
2	−5445.288	10,946.577	11,064.530	10,975.656	0.677	<0.001	<0.001
3	−5352.177	10,780.354	10,940.433	10,819.819	0.794	<0.001	0.0003
*4*	*−5039.056*	*10,174.111*	*10,376.316*	*10,223.962*	*0.894*	*<0.001*	*0.0146*
5	−5105.293	10,326.587	10,570.918	10,386.823	0.880	<0.001	0.5508

*Note*: The italicized 4th model is selected for subsequent analysis.

Abbreviations: AIC, Akaike Information Criteria; BIC, Bayesian Information Criteria; BLRT, Bootstrap Likelihood Ratio Test; LMR, Lo–Mendell–Rubin test; LPA, latent profile analysis; PHQ‐9, Patient Health Questionnaire‐9.

#### 3.4.2 Identified depression profiles

**FIGURE 2:**
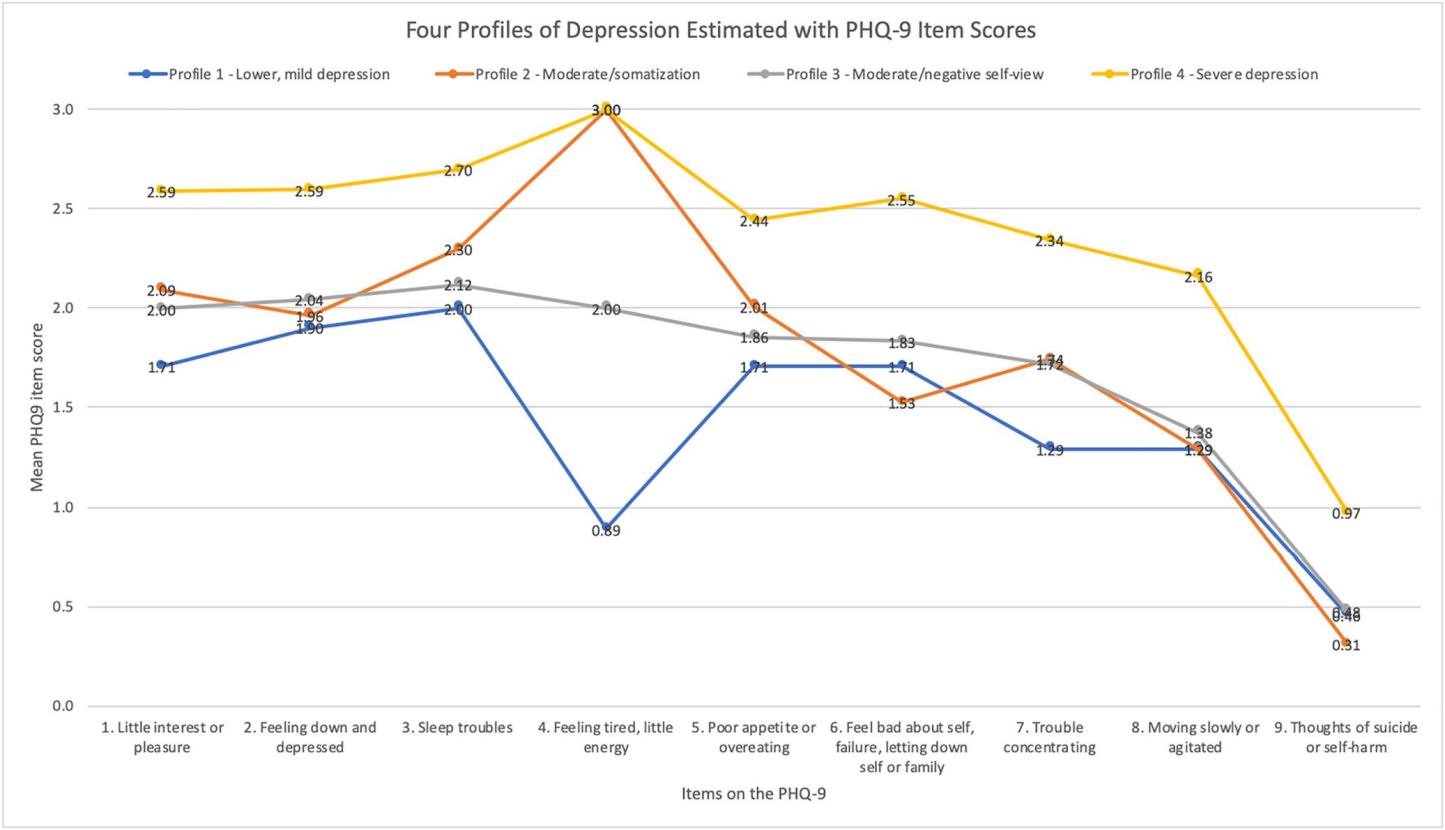
Latent profile analysis (LPA) on Patient Health Questionnaire‐9 (PHQ‐9; *N* = 499). This figure presents results from the LPA analysis with each profile plotted on a separate line. The mean values within each profile on each item of the PHQ‐9 are included above the data point

Profiles were found to be as follows (Figure 2 and Table 3):

**TABLE 3:** PHQ‐9 item mean scores per profile (*N* = 499)

PHQ items	Profiles of depression from LPA model			
	1: Mild depression (*n* = 104, 20.8%)	2: Moderate/somatization (*n* = 183, 36.7%)	3: Moderate/negative self‐view (*n* = 72, 14.4%)	4: Severe depression (*n* = 140, 28.1%)
1	1.71	2.09	2.00	2.59
2	1.90	1.96	2.04	2.59
3	2.00	2.30	2.12	2.70
4	0.89	3.00	2.00	3.00
5	1.71	2.01	1.86	2.44
6	1.71	1.53	1.83	2.55
7	1.29	1.74	1.72	2.34
8	1.29	1.29	1.38	2.16
9	0.46	0.31	0.48	0.97
Total	12.96	15.95	15.42	21.49

Abbreviations: LPA, latent profile analysis; PHQ‐9, Patient Health Questionnaire‐9.

**Profile 1:** lower, mild depression profile (*n* = 104, 20.8%); reported lowest mean item scores across seven of the nine items with only slightly higher scores on feeling bad about oneself and thoughts of self‐harm.

**Profile 2:** moderate/somatization profile (*n* = 183, 36.7%); reported higher mean item scores for items addressing anhedonia, sleeping problems, having little energy, and feeling like a failure.

**Profile 3:** moderate/negative self‐view profile (*n* = 72, 14.4%); reported moderate mean scores across the PHQ‐9 items. This group was found to have elevated mean items scores for items related to feeling down or hopeless, feeling bad about oneself, and psychomotor disturbances. In terms of PHQ‐9 overall severity, the somatization profile most closely aligned with the moderate/negative self‐view profile.

**Profile 4:** severe depression (*n* = 140, 28.1%); reported the highest mean item scores across all items in the PHQ‐9.

PHQ‐9 scores were significantly associated with profile membership, as expected (*F* [3, 495] = 199.33, *p* < 0.001). Individuals categorized as Profile 1 had significantly lower PHQ‐9 scores than each of the other three profiles, and Profile 4 individuals reported significantly higher scores than the other three. Individuals in Profiles 2 and 3 did not report significantly different scores.

#### 3.4.3 Association with covariates

The three‐step LPA approach was completed for all demographic variables and self‐report measure scores predicting likelihood of profile membership (Table 4). Anxiety assessed through the GAD‐7 was the most predictive of PHQ‐9 profile membership. When compared to Profile 1 (mild depression), a one‐point increase in reported GAD‐7 scores predicted an 8.8% increase in the likelihood of membership in Profile 3 (moderate/negative self‐view depression) and 32.9% increase in likelihood of membership in Profile 4 (severe depression). Profile membership was not associated with age of the participant, gender, or marital status.

**TABLE 4:** Multinomial logistic regression in Mplus, latent profile analysis three‐step approach results (*N* = 499)

Variable	Profile	*M* ± *SD*, *n* (%)	OR	*p*‐Value
Age	1: Mild depression	38.89 ± 10.31	ref.	‐
	2: Moderate/somatization	39.18 ± 11.21	0.997	0.849
	3: Moderate/negative self‐view	38.83 ± 9.79	0.997	0.891
	4: Severe depression	39.21 ± 9.75	0.999	0.986
Gender, *female*	1: Mild depression	63 (87.5%)	ref.	‐
	2: Moderate/somatization	94 (90.4%)	0.839	0.682
	3: Moderate/negative self‐view	168 (91.8%)	1.343	0.545
	4: Severe depression	134 (95.7%)	0.421	0.105
Married, *yes*	1: Mild depression	49 (68.1%)	ref.	‐
	2: Moderate/somatization	75 (74.3%)	0.739	0.373
	3: Moderate/negative self‐view	123 (67.2%)	1.039	0.897
	4: Severe depression	105 (75.0%)	0.710	0.284
Generalized Anxiety Disorder‐7	1: Mild depression	10.54 ± 5.23	ref.	‐
	2: Moderate/somatization	11.80 ± 4.06	1.065	0.069
	3: Moderate/negative self‐view	12.21 ± 4.17	1.088	0.008
	4: Severe depression	15.65 ± 3.99	1.329	<0.001

## 4 DISCUSSION

Results indicate modest support for the PHQ‐9 and its use among Hispanics in primary care for the purpose of depression screening and monitoring. Internal consistency reliability was poor, consistent with qualitative research on the measure which found patients felt the items did not adequately characterize their experience of symptoms of low mood (Malpass et al., 2016). Cronbach alpha coefficient assumes *tau*‐equivalence meaning that the statistic assumes all items have equal true scores (i.e., an assumption of equal factor loadings for all items in the model). Despite support for unidimensionality of the PHQ‐9, the lower alpha coefficient estimate is likely due to a lack of *tau*‐equivalence in the data (Dunn, Baguley, & Brunsden, 2014). Support for the unidimensional one‐factor structure of the PHQ‐9 was found with responses from Hispanic women from two community samples (Familiar et al., 2015; Merz, Malcarne, Roesch, Riley, & Sadler, 2011). PHQ‐9 data from a large sample of highly educated Mexican, female educators fit the one‐factor structure with higher reliability (⍺ = 0.89) but lower in subsamples within the study (*α* = 0.85 and 0.77; Familiar et al., 2015). No exploration was made of differing profiles of depression. Additionally, depression scores in this sample of Mexican educators were starkly different from the current study of treatment‐seeking Hispanic women who screened positive for depression in a clinical setting. Differences in the severity depression scores between the two sample could explain differences in obtained estimates of internal consistency reliability wherein the PHQ‐9 becomes less reliable when reported scores increase throughout the sample.

The single item related to fatigue had the greatest variation across profiles indicating it might be useful as a screening item. In Hispanic populations, especially women, careful attention to physical descriptions of symptoms could prove important to understanding depression severity and has implications for treatment. Over 100 studies have examined the PHQ‐9 for use in primary care and general medical settings (Kroenke, Spitzer, Williams, & Löwe, 2010), and severity of depression is routinely assessed by adding up scores for disparate symptoms to create a sum‐score, even though symptom variability among people diagnosed with depression is broad and collapsing all symptoms into a single sum‐score fails to characterize the unique combination for an individual (Fried & Nesse, 2015; Zimmerman, Ellison, Young, Chelminski, & Dalrymple, 2015). The negative correlations between error terms in the factor model (Figure 1) are likely representative of the varying profiles of depression. For example, the somatization item of “feeling tired or having little energy” varied greatly between profiles and was found to have error variance negatively correlated with other items in the model. The heterogeneity of item scores between the profiles likely resulted in a lower internal consistency reliability coefficient for the sample as a whole (*n* = 499).

In this large sample of treatment‐seeking Hispanics in primary care, we found distinct profiles of depression which indicate varying clinical presentations and reporting of depression. Capturing these “communities of symptoms” (Djelantik et al., 2019) which occur together helps characterize the manifestation of depression in a Hispanic population, especially among female patients with moderate depression (Profiles 2 and 3). In Profile 2, there was overlap among somatization symptoms. For example, “feeling tired or having little energy” and “trouble falling asleep or staying asleep, or sleeping too much” were strongly connected. Because these symptoms have overlapping characteristics and occurred with moderate depression overall, we consider their occurrence together as distinct indicators of the same phenomenon. In Profile 3, there was overlap among symptoms related to negative self‐view and psychomotor disturbances. For example, we found that the symptoms “feeling down, depressed, or hopeless” was strongly associated “feeling bad about yourself,” “trouble concentrating,” and “moving or speaking so slowly that other people could have noticed,” a community of symptoms found to cause significant impairment in functioning but which can be difficult to tease out in clinical practice (Fried & Nesse, 2015).

Findings further support existing knowledge about the correlation between depression and anxiety, especially among women, which extends previous research and clinical discussions suggesting a close, predictable relationship between the two disorders (Goldberg & Fawcett, 2012). This finding also corresponds to current themes in the refining of the classification of anxiety and depression which suggest sufficient similarity and overlap of symptoms to consider a clinical grouping reflecting this comorbidity or that these disorders may reflect different manifestations of the same condition (Andrews, Anderson, Slade, & Sunderland, 2008). Additionally, high rates of undiagnosed or untreated depression and anxiety among Hispanic women suggest significant burden to the health of Hispanics in the United States, for which genetic and environmental factors, chronic health issues, and socioeconomic stressors can precipitate or accentuate (Wassertheil‐Smoller et al., 2014).

The substantial symptom variation among individuals who all qualify for a single diagnosis has been previously examined and our findings further highlight the potential explanation for the difficulty in achieving treatment efficacy given the different depression profiles (Fried & Nesse, 2015). It is common practice to use measurement sum scores as a criteria for establishing depression severity and using algorithms to treat accordingly, especially in primary care (Manea, Gilbody, & McMillan, 2015). However, such broad assumptions may be unjustified because depression symptoms differ in their impact on impairment and functioning, and individuals with similar total severity scores can have very different syndromes (Cohen, Greenberg, & IsHak, 2013; Fried & Nesse, 2015).

Results from the current study highlight the importance of looking beyond depression measurement summary scores and examining specific symptoms experienced by patients during treatment. Inadequate evaluation of specific subsets of symptoms could lead to significant under identification of the disorder (Fried, 2017) and contribute to treatment disparities in Hispanic populations. Clinician focus on acute symptoms of mood without consideration of residual symptoms—including anxiety and psychomotor functioning—is common and increases the likelihood of relapse (Trivedi, 2004). Some specific clinical characteristics may inform the choice between medication and psychotherapy, the selection of specific medication, or the selection of a specific psychotherapy (Simon & Perlis, 2010). A thorough assessment of all symptoms and their causal associations is necessary in order to achieve lasting remission and represents an initial step toward personalized treatment of depression that recognizes the heterogeneity of the disorder (Fried & Nesse, 2015; Trivedi, 2009).

Study results should be interpreted in light of its limitations. First, the study sample was primarily women, who generally report greater symptoms of anxiety/somatization compared to men (Kornstein et al., 2000). A majority of the participants were also Spanish‐speaking, a variable that was not controlled for in the analyses. However, prior research among Hispanic women support a similar one‐factor structure of the PHQ‐9 with equivalent response patterns among English and Spanish speakers (Merz, Malcarne, Roesch, Riley, & Sadler, 2011). Analyses with more heterogeneous samples or samples composed of primarily men warrant further investigation. Additionally, examining profiles of depression symptoms to better understand the various clinical presentations that may exist would benefit from multiple assessments including both self‐report and clinician‐rated. However, it should be noted that the utility of identifying subtypes of depression may be limited given that the extent to which proposed subtypes or even individual symptoms change over time is uncertain (Ulbricht, Rothschild, & Lapane, 2016). The sample was obtained through convenience sampling and was from two intervention studies of Hispanic patients who met diagnostic criteria for depression in an urban primary care setting in Texas, hence generalizability of results may be limited.

In conclusion, applying psychometric approaches like CFA and LPA to the most widely used depression screening and monitoring measure in primary care can yield important insights at the level of the individual because it allows for examination of relationships between symptoms and underlying dimensions. The described profiles identified through person‐centered statistical techniques may be meaningful in clinical assessment to tease out the burden of symptoms and personalize treatment accordingly. And while the present findings about profiles may be too complex to have acceptable clinical utility, a focus on varying manifestations of depression can be useful for clinicians to differentiate profiles from sum scores in conjunction with decision support tools when indicated.

## CONFLICT OF INTERESTS

The authors declare that they have no conflict of interest.
